# Allosteric inhibition of aminopeptidase N functions related to tumor growth and virus infection

**DOI:** 10.1038/srep46045

**Published:** 2017-04-10

**Authors:** César Santiago, Gaurav Mudgal, Juan Reguera, Rosario Recacha, Sébastien Albrecht, Luis Enjuanes, José M. Casasnovas

**Affiliations:** 1 Centro Nacional de Biotecnología (CNB-CSIC), Darwin 3, Campus Universidad Autónoma de Madrid, 28049 Madrid, Spain; 2 Department of Biotechnology, Institute of Engineering and Technology, Mangalayatan University, 33rd Milestone, Beswan, Aligarh, UP, India-202145; 3 INSERM, Aix-Marseille Université, CNRS, AFMB UMR 7257, 163 avenue de Luminy, 13288 Marseille, France; 4 Latvian Institute of Organic Synthesis, Aizkraukles 21, Riga LV-1006, Latvia; 5 Laboratoire de Chimie Organique et Bioorganique, Ecole Nationale Supérieure de Chimie Mulhouse, Université Haute-Alsace, 68093 Mulhouse, France

## Abstract

Cell surface aminopeptidase N (APN) is a membrane-bound ectoenzyme that hydrolyzes proteins and peptides and regulates numerous cell functions. APN participates in tumor cell expansion and motility, and is a target for cancer therapies. Small drugs that bind to the APN active site inhibit catalysis and suppress tumor growth. APN is also a major cell entry receptor for coronavirus, which binds to a region distant from the active site. Three crystal structures that we determined of human and pig APN ectodomains defined the dynamic conformation of the protein. These structures offered snapshots of closed, intermediate and open APN, which represent distinct functional states. Coronavirus envelope proteins specifically recognized the open APN form, prevented ectodomain progression to the closed form and substrate hydrolysis. In addition, drugs that bind the active site inhibited both coronavirus binding to cell surface APN and infection; the drugs probably hindered APN transition to the virus-specific open form. We conclude that allosteric inhibition of APN functions occurs by ligand suppression of ectodomain motions necessary for catalysis and virus cell entry, as validated by locking APN with disulfides. Blocking APN dynamics can thus be a valuable approach to development of drugs that target this ectoenzyme.

Aminopeptidases are widely distributed among plants and animals, and catalyze amino acid cleavage from the N terminus of proteins and peptides^1^. Numerous aminopeptidases have been identified and grouped in several families^2^. They can be soluble or membrane-bound, they bear metal ions at the active site and are also known as metallopeptidases or metalloproteases. Zinc is the most common metal ion in aminopeptidases, and one or two zinc atoms can be present in the active site^1^. Aminopeptidase N (APN, CD13) belongs to the M1 family of zinc metallopeptidases^3^, also termed gluzincins, which comprises enzymes of medical interest for infectious and autoimmune diseases, cancer and hypertension^4^,^5^,^6^,^7^,^8^,^9^,^10^.

APN is associated with various cell functions and diseases, and has been termed the “moonlighting enzyme”^3^. It is a zinc-dependent metallopeptidase that hydrolyzes the N terminus of biological peptides and extracellular matrix proteins. APN-mediated peptide hydrolysis and activation is important for regulation of several physiological processes, such as the control of blood pressure (angiotensins) or cell chemotaxis. In addition, APN is linked to cell motility and tumor metastasis. APN is overexpressed in the neovasculature, including tumor blood vessels; its relevance in angiogenesis was discovered using NGR peptides and was later confirmed in animal models^7^,^11^. Peptides and inhibitors target APN overexpressed in tumors, and prevent tumor cell growth and invasion^12^,^13^; drugs that bind to the active site of this protein have been developed to treat tumors and some are in clinical trials^10^. In addition to its association with angiogenesis and cancer, APN is a major coronavirus (CoV) cell entry receptor^4^,^5^,^14^. CoV recognition of APN is species-specific, and specificity is associated with N-linked glycosylation in the APN protein^15^,^16^. We defined how some CoV recognize their APN receptor and the basis for virus-receptor binding specificity^16^.

Mammalian APN are type II membrane-bound ectoenzymes that dimerize on the cell surface^3^,^17^. They are composed of a short cytoplasmic domain, followed by the transmembrane domain and an extracellular ectodomain with ~930 amino acid residues and several N-linked and O-linked glycosylations^3^,^18^. The APN ectodomain is composed of four domains, domain I to IV^16^,^19^, connected to the membrane by a short linker or stalk^3^,^18^. The three N-terminal domains I-III are more similar to homologous domains of M1 family members, whereas domain IV is more divergent^3^. Domain II has a thermolysin-like fold and contains the zinc-coordination residues conserved in the active site of gluzincins, two histidines in the HEXXH motif, and a glutamic acid ~24 residues distal from that motif^2^,^16^,^19^. Several crystal structures of APN in complex with peptides and drugs showed how substrates bind to the active site, providing clues to peptide hydrolysis^19^,^20^,^21^. The human and pig APN ectodomains share 79% residue identity, domain architecture and dimerization^16^,^19^. The C-terminal domain IV is the largest APN domain and, according to the crystal structures, mediates protein dimerization^16^,^19^. Porcine CoV recognize a membrane-distal domain IV region, which includes an N-linked glycan in pig APN, as well as a small cavity formed by domain II and IV^16^. The mode by which other CoV recognize the angiotensin-converting enzyme 2 (ACE2) or dipeptidylpeptidase 4 (DPP4) is distinct from APN^22^, although a glycan N-linked to DPP4 is also involved in the interaction.

Structures of the M1 metallopeptidases tricorn-interacting factor F3 and the endoplasmic reticulum aminopeptidase-1 (ERAP-1) indicate that M1 family members can adopt diverse conformations^23^,^24^. F3 structures define a closed, an intermediate and an open state, based on accessibility of the catalytic site in domain II, which is determined by the position of domain IV relative to domain II. It is proposed that substrates gain access to the active site in the open state and that the enzyme must close for peptide hydrolysis^20^,^23^,^24^. Nonetheless, the role in catalysis of the dynamic transition between states has not been determined. Functional assays using monoclonal antibodies (mAb) indicated a dynamic conformation for cell surface APN; the MY7 mAb recognized a subset of APN molecules, and the presence of its epitope in HL60 cells varied with substrates and/or inhibitors^25^. Antibody binding did not alter protein dimerization, but pointed to changes in APN ectodomain conformation on the cell surface^25^.

We determined several crystal structures of dimeric human and porcine APN (hAPN and pAPN) ectodomains, which reveal the dynamic conformation of this protein. A dimer is preserved in all structures. The receptor-binding domain (RBD) of a porcine CoV bound specifically to the open pAPN form and inhibited catalysis, probably by preventing APN dynamics and closure. In contrast, drugs that bound to the active site, which likely maintained a closed protein, inhibited CoV protein binding and infection. Blocking APN ectodomain motion with disulfide bonds inhibited its functions. Thus, our results show the importance of APN dynamics in catalysis and virus infection.

## Results

### The APN ectodomain structure

As the APN protein is a type II membrane protein, ectodomain expression required deletion of the N-terminal cytoplasmic and transmembrane domains, and introduction of a secretion signal sequence, as well as a hemagglutinin (HA) tag to allow protein detection and purification (Supplementary Fig. S1a). As the N-terminal and middle portions of the hAPN and pAPN ectodomains are heavily glycosylated, we produced them in CHO cells (see Methods). The purified proteins generated distinct crystal forms under different crystallization conditions (Table 1 and Methods).

**Figure 1:**
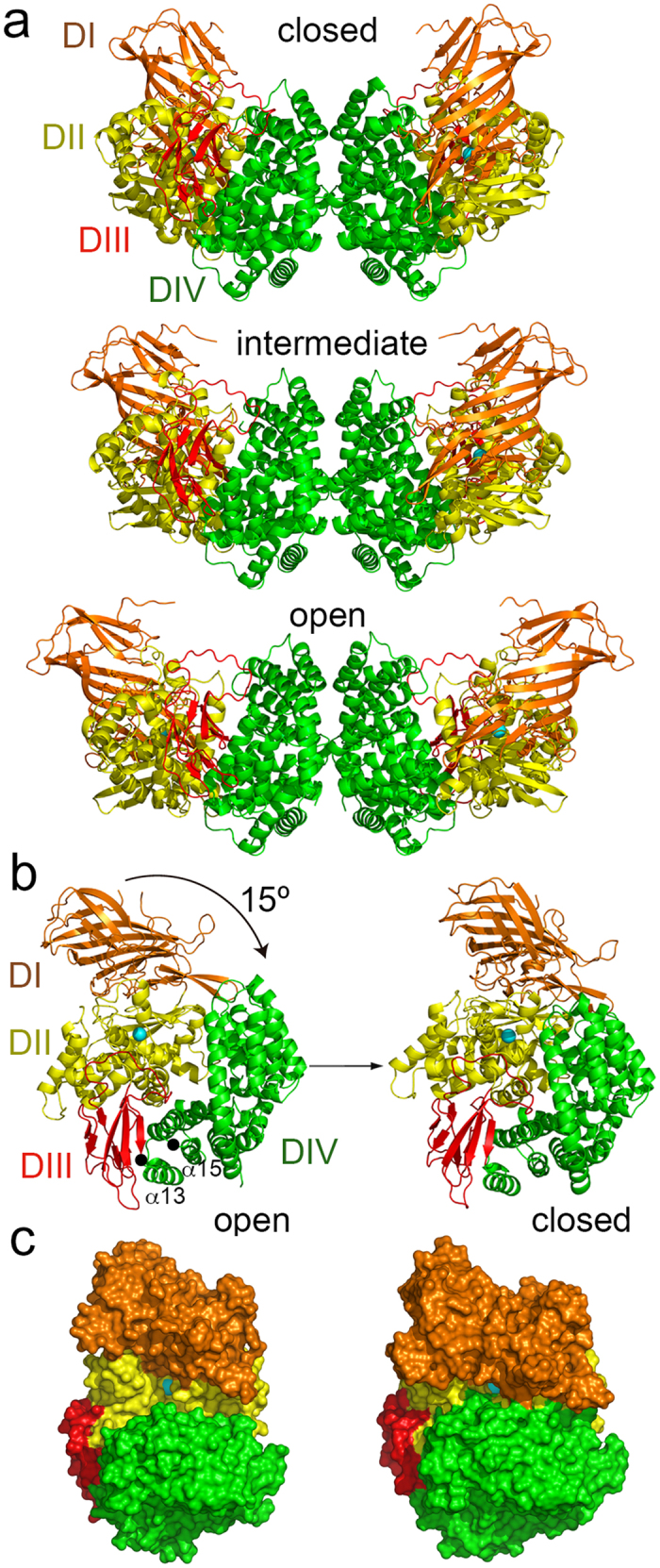
The dynamic conformation of mammalian APN ectodomains. (**a**) Ribbon representation of the closed (pAPN), intermediate (hAPN) and open (pAPN-RBD, PDB code 4F5C) dimeric APN structures (Table 1). The APN domains colored in orange (domain I, DI), yellow (domain II, DII), red (domain III, DIII) and green (domain IV, DIV). N-linked glycans omitted. (**b**) Interdomain movement between the open and closed APN conformations. The arrow indicates the swing movement of the domain I-II-III module toward domain IV after ectodomain closure in each monomer of the APN dimer, with domain IV fixed by dimerization. The hinge residues at the N terminus of α13 and α15 in domain IV are marked with black dots. See also Supplementary Video S1. (**c**) Surface representation of the open and closed APN. Front view of the active site, with the zinc ion in domain II as a cyan sphere.

In the past we reported a pAPN ectodomain crystal structure in complex with a CoV spike (S) fragment (PDB code 4F5C)^16^, here we show three new structures for APN (Table 1). In the four structures, the N-terminal HA tag and ~30 ectodomain residues were very disordered, indicating a large degree of flexibility in the membrane proximal polypeptide. The ectodomains have a hook-like conformation formed by domain I to IV and contained a zinc ion at the active site in domain II (Fig. 1). The exposed convex side of domain IV mediates similar protein dimerization in the distinct crystals. Approximately 950 Å^2^ of each monomer is buried at the dimer interface (Table 2), indicative of a stable protein-protein interaction. Domain IV is the largest APN domain and the most divergent in the M1 aminopeptidase family. In APN, domain IV has seven helix-turn-helix HEAT repeats and a single ARM repeat formed by three alpha helices (α25-α27). The ARM repeat is the most variable domain IV region in the hAPN and pAPN structures, and can contact the peptide substrate bound to the active site (see below).

### Dynamic conformation of the APN ectodomain

Although the dimeric assembly of human and pig APN ectodomains was preserved in various crystals, the conformation of each monomer differed among crystal forms, such that the distance between the N-terminal region of the ectodomains that formed the dimer varied from 95 to 131 Å in the structures (Table 2 and Supplementary Fig. S1b). Each crystal captured a single APN conformation, with all the monomers in the same form. These structures identified three distinct APN conformations, based on active site accessibility, which we termed closed, intermediate and open forms (Fig. 1a). As reported for other M1 family members^23^,^24^, the observed APN structural diversity indicated ectodomain dynamics in solution and on the cell surface.

The active site accessibility at domain II differed among crystal forms because of interdomain adjustments in the APN. The contacts between domain IV and other domains in the monomers varied among the structures, whereas the domain IV-IV buried surfaces in each monomer at the dimerization interfaces were preserved (Table 2). Domain IV contacts with domain I or III changed markedly less (~100–200 Å^2^ buried surface) than with domain II (1000 Å^2^); domain II-IV interaction thus mainly stabilized the closed APN conformation. There were no notable differences in the other interdomain contacts in the distinct APN forms (Table 2). Domains I-II are distant from domain IV in the open conformation of the APN monomer (Fig. 1a,b), where the zinc ion at the catalytic site is more accessible to the solvent (Fig. 1c). The domain I to III module swings 15° toward domain IV, closing the active site (Fig. 1b,c). The hAPN structure adopts an intermediate conformation in the crystals (Fig. 1a); the distance between the N terminus of each monomer in the dimer is 116 Å, and the angle difference of domain II with the closed conformation is ~6° (Table 2). Other crystal structures of human and pig APN showed closed and intermediate conformations^19^,^21^, respectively, similar to the forms reported here.

APN dimerization mediated by domain IV-IV interactions is preserved among open, intermediate and closed ectodomains (Fig. 1a and Table 2). On the cell surface, the domain I to III module must swing over domain IV, which is fixed by dimerization (Fig. 1b, Supplementary Video S1). The module movement must be facilitated by the flexible ~30-residue polypeptide that links domain I to the transmembrane domain (Supplementary Fig. S1a), although polypeptide length probably limits the interdomain movement shown here with APN (15°), which is less pronounced than that reported for ERAP-1 (22°, determined as in Table 2). The type of interdomain movement also differs between ERAP-1 and the APN. The domain III-IV module moves together relative to domain I-II in ERAP-1, whereas domain I to III swings over domain IV in the APN. In addition, the ERAP-1 hinge region is at the domain III N terminus, whereas that of APN is in the domain IV N terminus. Domains I, II and III can pivot at the beginning of the first (α13) or third (α15) domain IV helix, which are perpendicular to the swing angle (Fig. 1b). These differences in APN motion compared to other aminopeptidases are probably related to dimer formation, which is not observed in other M1 family members.

### APN dynamics in catalysis and CoV binding

The domain II buried surface increases due to its interaction with domain IV when the conformation changes from open to closed (Table 2), thus reducing accessibility of the active site cavity (Fig. 1c). M1 aminopeptidase dynamics is thought necessary for catalysis, and the closed conformation is considered the active form^20^,^24^, although it remains unclear how this structural switch connects to peptide processing. Conformation of the domain II residues that participate in zinc coordination and peptide hydrolysis was conserved among the different APN structures shown here (Supplementary Fig. S2).

**Figure 2:**
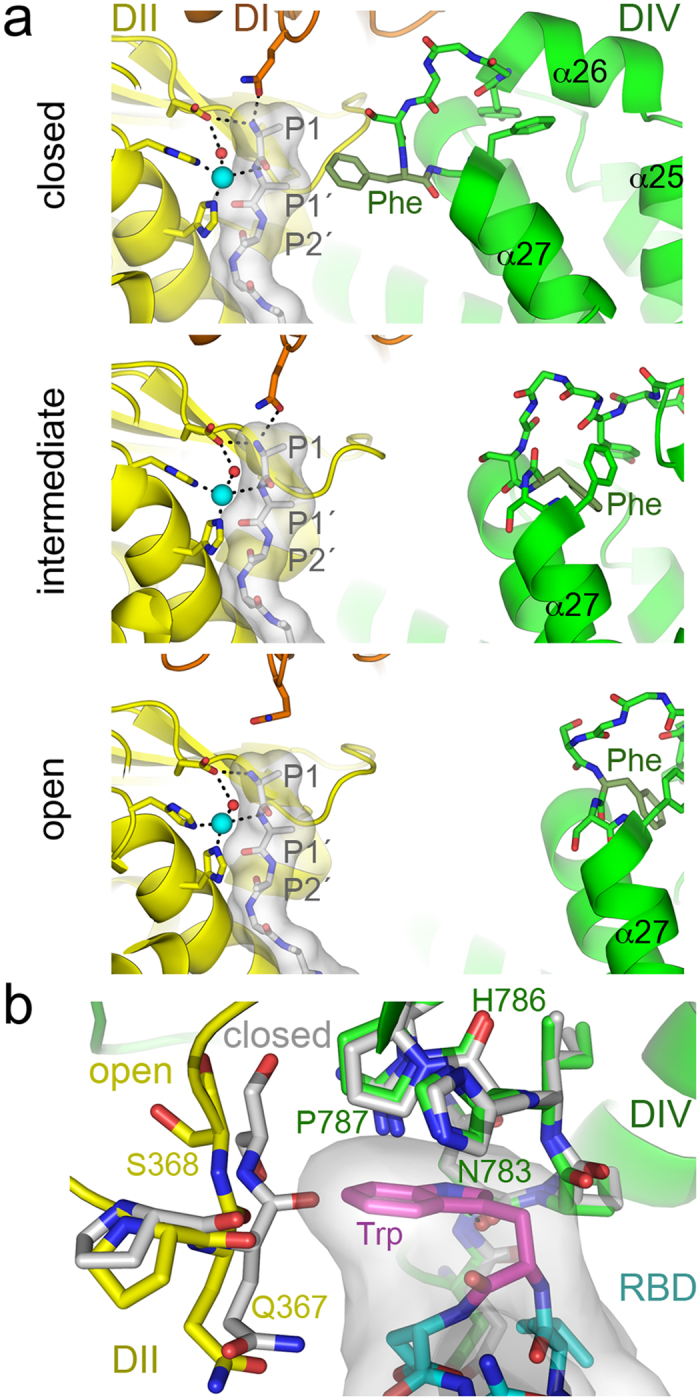
APN dynamics in catalysis and CoV recognition. (**a**) The active site of the closed, intermediate and open APN during peptide hydrolysis. The active site at domain II (DII, yellow) contains a modeled poly-alanine peptide coordinated to the zinc ion (cyan sphere). Side chains of APN active site residues are shown with sticks, whereas the poly-alanine is shown as a gray surface with residues as sticks (carbons in grey). N-terminal peptide residues (P1-P1′-P2′) are labeled. Nitrogens, blue; oxygens, red; hydrogen bonds are dashed lines. The helices of the ARM repeat (α25-α27) in domain IV (DIV, green) with the phenylalanine residue that contacts the peptide in the closed conformation (Phe893 in pAPN) are labeled. The crystal structure of the poly-alanine bound to the pAPN (PDB code 4HOM) was used to model it in the active site of closed, intermediate and open structures by structural superposition based on domain II. (**b**) Conformation of the CoV binding cavity at the domain II-IV interface in the closed and open pAPN structures. Structures were superposed based on domain IV. Ribbon diagrams of the open pAPN in complex with the porcine CoV RBD (Supplementary Fig. S1b), with residues that contact the RBD in sticks with carbons in yellow (domain II) and green (domain IV). The same residues are shown for the superposed closed structure (carbons in grey). The RBD motif that penetrates the pAPN cavity is shown with a grey surface and with residues as sticks (carbons in cyan or in magenta for Trp).

Crystal structures are reported for mammalian APN in complex with substrates, in both intermediate (pAPN) and closed conformations (hAPN)^19^,^21^. To determine how domain movement contributes to peptide processing, we modeled a pAPN-bound poly-Ala peptide in the active site of closed, intermediate and open APN (Fig. 2a). In the closed pAPN, the side chain of a phenylalanine (Phe893) at domain IV was placed at about 4.5 Å from the hydrolyzable peptide bond, whose carbonyl group is coordinated to the zinc ion. The phenylalanine was located in the loop that connects α26 and α27 in the single domain IV ARM repeat of human and pig APN (Fig. 2a); it penetrated the active site groove in the closed conformation and locked the peptide, ready for hydrolysis. Domain IV residues that precede Phe893 in the α26-α27 loop contacted domain II in the closed pAPN. A similar loop conformation is seen in a closed hAPN structure (PDB code 4FYS)^19^.

The phenylalanine side chain in closed APN probably hinders peptide release or translocation for further processing after P1 hydrolysis. It is likely that binding of the P1′ residue to the zinc ion required removal of the phenylalanine plug by domain II displacement away from domain IV. The phenylalanine adopted a distinct conformation in the intermediate and open APN conformations (Fig. 2a). Domain II movement was accompanied by a conformational change of the α26-α27 loop in domain IV (Supplementary Video S1), which became more solvent-exposed; the phenylalanine side chain faced into domain IV in the intermediate and open conformations and the peptide plug was removed from the active site. These changes would facilitate release of the N-terminal residue after hydrolysis. The small interdomain movement of the intermediate APN structure would be sufficient for peptide processing (Fig. 2a).

We previously described in detail the CoV spike RBD-pAPN binding interface^16^. The porcine CoV spike RBD binds to a pAPN region that is distant from the catalytic site (Supplementary Fig. S1b). A critical CoV receptor-binding motif, which bears an exposed tryptophan, penetrates a narrow cavity formed by domain II and IV (Fig. 2b)^16^. The tryptophan aromatic side chain stacks onto pAPN domain IV residues His786-Pro787, and is trapped by domain IV residues Asn783-Pro787 on one side and domain II residues Gln367-Ser368 on the other (Fig. 2b). The main chain of domain II residues is in close contact (3.9 Å) with the tryptophan side chain, and its imino nitrogen forms a hydrogen bond with the domain IV Asn783 main chain carbonyl. Domain IV-based superposition of the open pAPN with bound RBD and that of closed pAPN showed a shift in the domain II main chain region that contacts the RBD; this region collides (<3.0 Å) with the CoV tryptophan (Fig. 2b). Closing of the ectodomain would hinder penetration of the viral tryptophan between the pAPN domain II and IV.

### Allosteric inhibition of APN catalysis by CoV binding to the ectodomain

**Figure 3:**
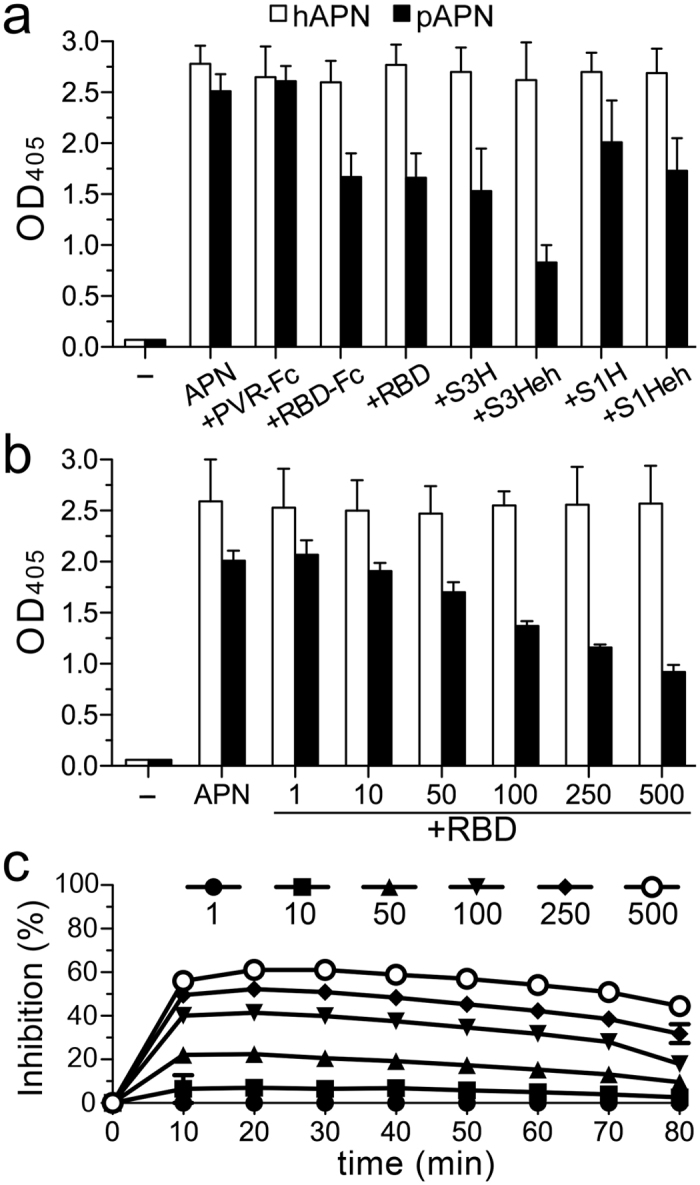
Allosteric inhibition of APN catalysis by CoV S proteins. APN catalysis was monitored as optical density (OD) with soluble hAPN and pAPN ectodomains (see Methods). (**a**) Activity determined with pAPN and hAPN (0.04 μM) alone or with porcine CoV S proteins untreated (S1H, S3H) or endoglycosidase H-treated (S1Heh, S3Heh), or with the TGEV RBD fragment; all these proteins bind specifically to pAPN^16^,^35^. The unrelated PVR-Fc protein was used as control. Sample without APN (−). (**b**) Activity determined at 60 min with increasing RBD:APN molar ratios. (**c**) Inhibition of pAPN catalytic activity by the TGEV RBD over time. Inhibition was computed according to the OD in b.

Our data suggested that CoV binding to APN would lock the protein in its open conformation (Fig. 2b), preventing the ectodomain movement probably necessary for peptide hydrolysis (Fig. 2a). We analyzed the catalytic activity of soluble human and pig APN ectodomains in the presence of porcine CoV S fragments bearing the RBD (Fig. 3). The soluble S proteins specifically inhibited pAPN-mediated catalysis, measured as the hydrolysis of the L-pNA substrate, and had no effect on hAPN activity. The TGEV (transmissible gastroenteritis coronavirus) spike does not bind hAPN because it lacks the N-linked glycan recognized by porcine CoV in pAPN^15^,^16^.

The isolated RBD was sufficient to inhibit pAPN catalysis (Fig. 3a); inhibition was dependent on RBD concentration. A high RBD:pAPN ratio was needed to achieve maximum inhibition (50–60%; Fig. 3b), which decreased slowly after 30 min (Fig. 3c), probably due to substrate hydrolysis during RBD dissociation and binding to APN over time. The RBD can thus prevent catalysis in only a fraction of pAPN molecules, probably those in the open form. The conformation of catalytically active pAPN would not allow RBD binding. The results indicate that the open APN is catalytically inactive, and that blocking APN dynamics impairs catalysis.

### Drugs that bind the catalytic site inhibit CoV binding to APN

Non-hydrolyzable drugs that bind the APN catalytic site inhibit catalysis and prevent angiogenesis and tumor growth^7^,^10^,^26^,^27^. They appear to restrict ectodomain conformational changes, as shown by reduction in the number of some APN conformation-specific mAb epitopes^25^. On the cell surface, active site epitopes recognized by the MY7 mAb decrease in the presence of actinonin, which indicates APN closure. Crystal structures of M1 aminopeptidases in complex with these drugs show preferential adoption of a closed state^19^,^20^,^24^,^28^. Drug binding would thus not only compete with substrates for active site binding, but might also restrict the aminopeptidase dynamics needed for peptide processing.

**Figure 4:**
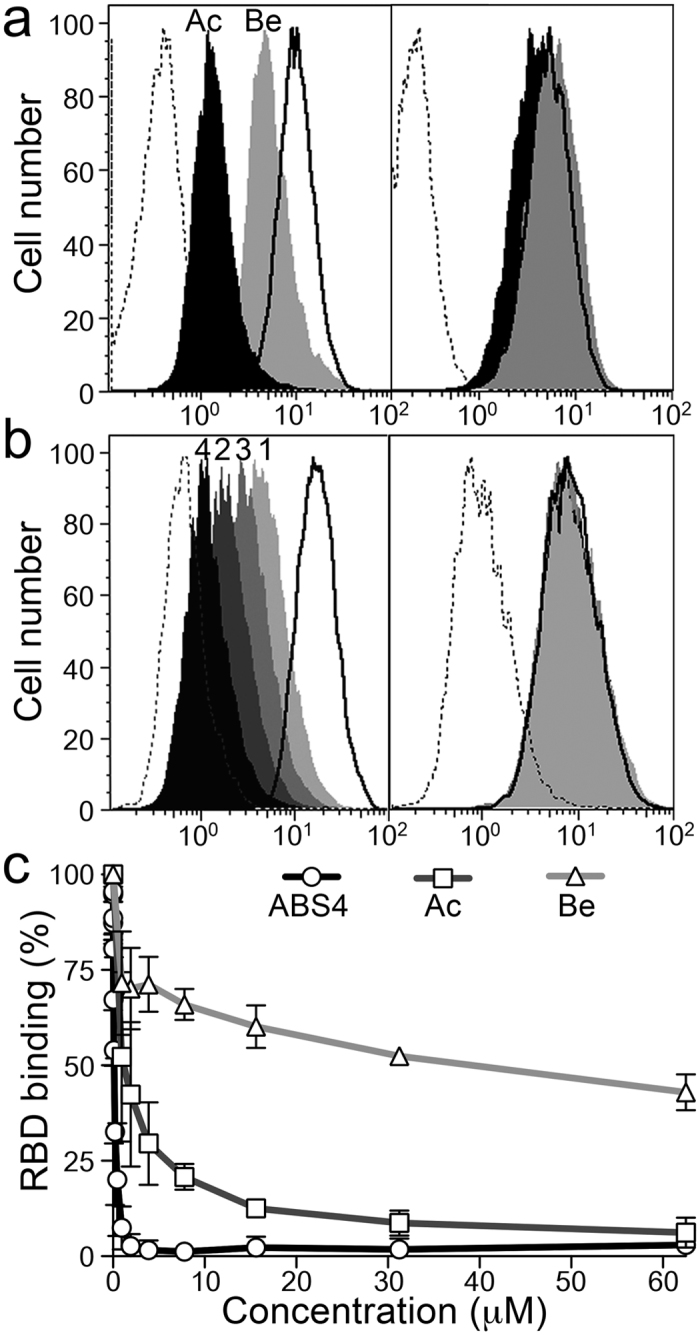
Drugs that bind the catalytic site inhibit CoV binding to APN. (**a,b**) Flow cytometry to monitor TGEV RBD-Fc protein (~1 μg/ml) binding to CHO cells expressing pAPN (left panels) or a pAPN catalytic mutant (right panels). The mutant (pAPN-HH/AA) lacks the two histidines that coordinate the zinc ion (Supplementary Fig. S2). Histograms recorded with the RBD-Fc protein alone (solid line histograms) or with pAPN active site-binding drugs (filled histograms) are shown. Control unrelated Fc fusion protein, dashed line histogram. Overlay plots of histograms for samples alone or with 500 μM bestatin (Be; gray) or actinonin (Ac; black) in (**a**); samples with synthetic APN-binding drugs ABS1, 3, 2, and 4 (light gray to black; as numbered) in (**b**). Approximate pAPN Ki values for bestatin and actinonin are 4 and 1 μM^29^, respectively, and 40, 7, 19 and 0.06 nM for ABS compounds 1 to 4^30^. Described in Supplementary Fig. S3a. (**c**) Relative RBD-Fc binding to pAPN-expressing BHK cells, alone or with increasing concentrations of bestatin, actinonin and ABS4. Mean fluorescence intensity computed by flow cytometry was used to calculate RBD binding ratios (see Methods). Mean ± SD (*n* ≥ 3).

The structure of the pAPN-RBD complex indicates that porcine CoV would be specific for the open conformation (Fig. 2b). Restriction of APN ectodomain opening by active site-binding drugs would thus have an allosteric effect on CoV binding. To test this hypothesis, we studied TGEV RBD binding to cell surface pAPN in the presence of drugs that bind to the active site (Fig. 4). In flow cytometry, we determined the binding of an RBD-Fc fusion protein to cells that expressed pAPN or an active site mutant (pAPN-HH/AA), alone or with various drugs (Fig. 4a,b). We analyzed the effect of the natural APN inhibitors actinonin and bestatin^29^; both reduced RBD-Fc binding to cell surface pAPN (Fig. 4a, left). We then evaluated four synthetic amino-benzosuberone (ABS) derivatives that bind with high affinity and selectivity to APN (Supplementary Fig. S3a)^30^; all four ABS molecules prevented RBD binding to pAPN and its effectiveness increased with APN-binding affinity (Fig. 4b, left). The bulkier ABS2 and ABS4 compounds, which contain a phenyl group and bind with the highest affinity to APN, more efficiently blocked binding of the TGEV RBD to pAPN on the cell surface.

The inhibitory molecules bind to the APN active site^19^,^21^, which is distant from the APN region recognized by CoV (Supplementary Fig. S1b). To further determine whether the inhibitory effect was linked to drug binding to the pAPN active site, we analyzed RBD binding to the pAPN-HH/AA mutant, which lacks the two histidines (H383 and H387) that coordinate the zinc ion (Supplementary Fig. S2). Staining for the RBD-Fc protein was similar in cells expressing the mutant, alone or with the drugs (Fig. 4a right and b right), which showed that compound binding to the pAPN active site was necessary to prevent RBD binding to a distant site. In addition, inhibition of RBD binding to pAPN was drug concentration-dependent (Fig. 4c), and the amount of compound needed to reach 50% inhibition (IC_50_) decreased with compound affinity for APN (~30 μM for bestatin (Ki ~4 μM), ~1 μM for actinonin (Ki ~1 μM), ~0.1 μM for ABS4 (Ki ~0.06 nM)). These results show that drugs that bind to the active site cause allosteric inhibition of TGEV RBD binding to pAPN, probably by restricting APN ectodomain dynamics and its transition to the CoV-specific open form.

### Anti-tumor APN-binding drugs inhibit CoV cell infection

**Figure 5:**
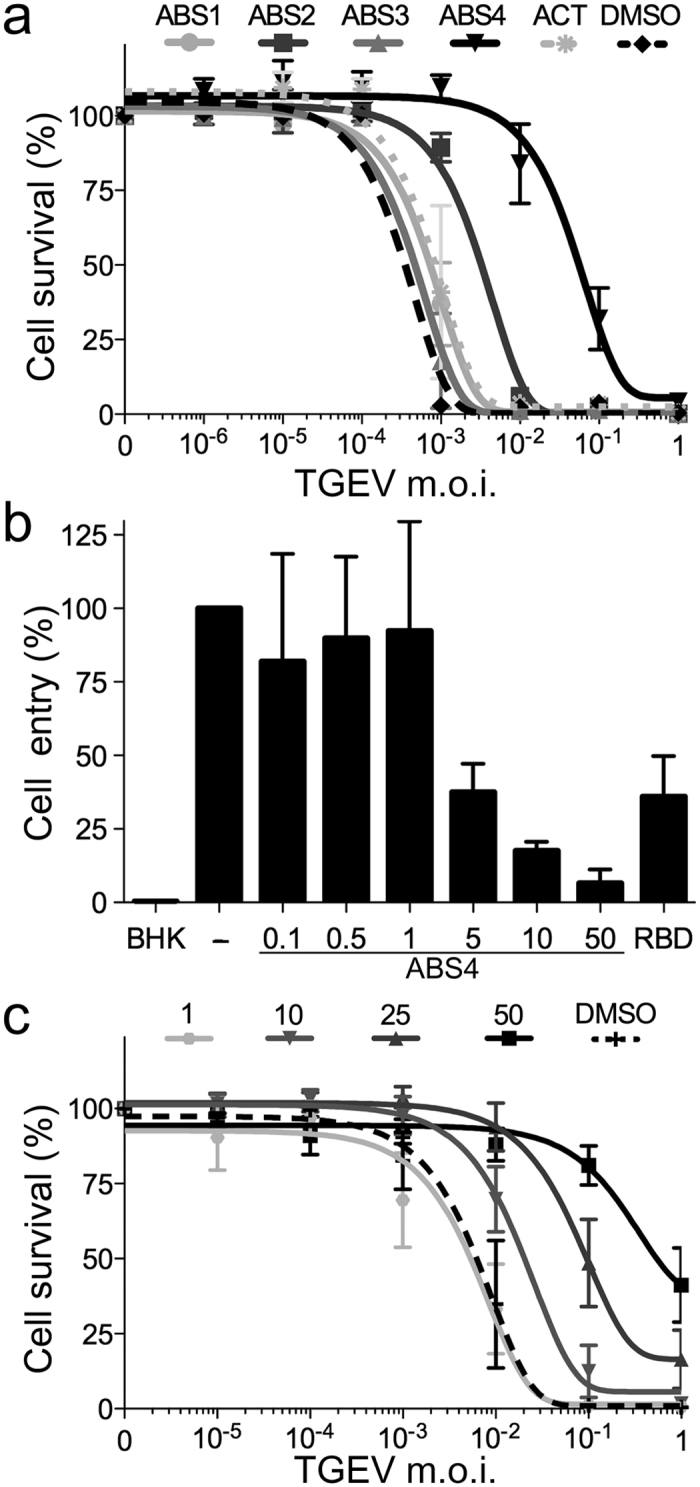
Catalytic site-binding drugs inhibit CoV cell infection. (**a**) Inhibition of TGEV infection by various pAPN-binding drugs. Relative survival of ST cells infected with TGEV at different m.o.i., treated with 50 μM ABS1-4, actinonin (Ac) or DMSO (Methods). See Supplementary Fig. S3b. Mean ± SEM (*n* = 3). (**b**) Inhibition of TGEV cell entry by ABS4. Virus RNA was quantified by qRT-PCR 6 h post-infection, in samples without inhibitor (−), with ABS4 as indicated (μM) or with TGEV RBD-Fc (40 μg/ml) as positive control (RBD). Background signal determined with uninfected BHK cells (BHK). Mean ± SEM (*n* = 3). (**c**) Inhibition of TGEV cell infection with increasing ABS4 concentrations. ST cell survival was determined as in a. Mean ± SEM (*n* = 4).

APN catalytic activity is not necessary for CoV cell entry and infection^31^. We nonetheless found that active site-binding molecules hindered CoV S protein binding and might inhibit virus infection. Studies with low affinity binding drugs such as bestatin show no reduction in TGEV infection^31^. Virus particles have high receptor-binding avidity, and these drugs might not have sufficient affinity to maintain most APN molecules closed. The selective compounds ABS1-4 have high affinity for APN and, at 1–10 μM concentrations, inhibit capillary tube formation in cell cultures, with no cytotoxicity^27^. In our cultures, we observed no toxicity at ABS concentrations <100 μM (not shown). We therefore analyzed the TGEV-mediated cytopathic effect for each of the four ABS molecules and actinonin at a 50 μM concentration and monitored inhibition of virus infection with ABS4 (2 log) and ABS2 (1 log) (Fig. 5a); at the same concentration, the lower-affinity ABS1 and ABS3 compounds or actinonin did not inhibit. ABS4 has a bromo substituent that is predicted to interact with the phenylalanine that plugs the substrate in the closed conformation^32^; this interaction likely helped maintain the closed ectodomain and efficiently prevented virus binding.

TGEV is a representative, extensively studied animal CoV that use pAPN for cell entry^4^,^14^. To further determine whether ABS4 inhibition of virus infection was linked to cell entry, we analyzed TGEV replication at 6 h post-infection and found that virus entry decreased with the ABS4 concentration (Fig. 5b). ABS4 addition after virus absorption at 4 °C did not inhibit virus growth (not shown), which indicates that it prevented virus binding to cells. In addition, we studied the effect of ABS4 concentration on TGEV cell infection, and we observed that the TGEV cytopathic effect was reduced and cell survival increased at higher ABS4 concentrations (Fig. 5c). ABS compounds are selective for APN molecules and designed to inhibit APN catalytic activity and tumor growth; here we show that they also prevent CoV cell infections.

### Restricting APN dynamics inhibits function

**Figure 6:**
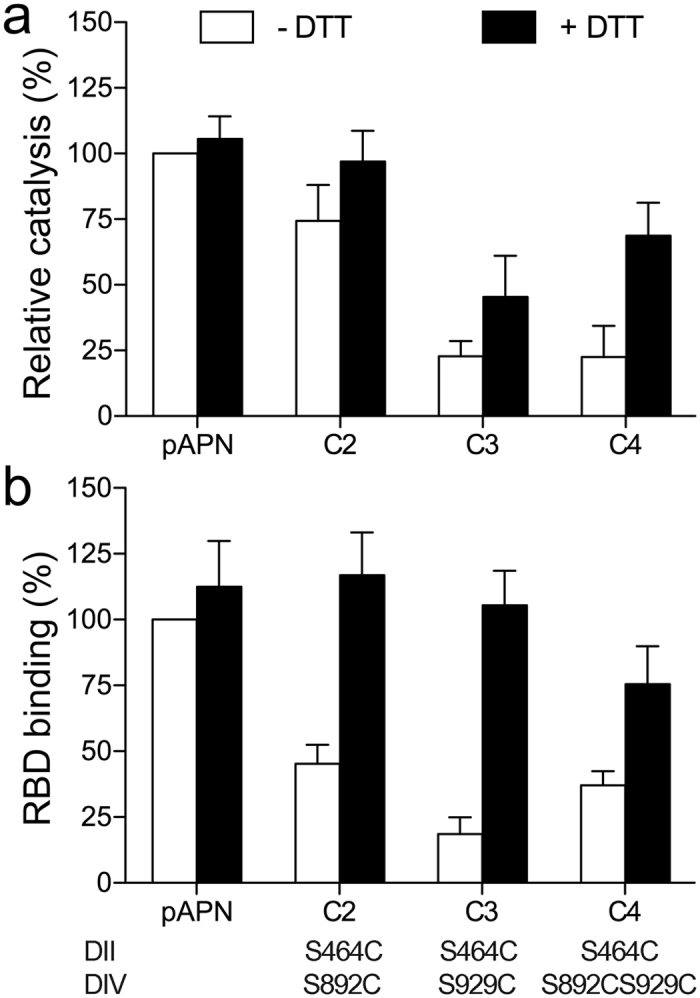
Locking the pAPN closed form with disulfide bonds inhibits its functions. Relative pAPN catalysis (**a**) and TGEV RBD binding (**b**) activity of native or pAPN-cysteine mutants (C2-C4) expressed on the 293T cell surface. Cells were incubated alone or with DTT (5 mM; 30 min, 37 °C) before assays. Values expressed as a percentage of wild type pAPN values in the absence of DTT. Activities were normalized to the amount of cell surface protein expressed as monitored by flow cytometry (see Methods). Catalysis was determined at 60 min as absorbance at OD405 nm (see Fig. 3 and Methods). Relative RBD-Fc binding to transfected cells determined from mean fluorescence intensity computed by flow cytometry as in Fig. 4c. Domain II and IV residues replaced by cysteine are indicated at bottom (see Supplementary Fig. S4). Mean ± SD (*n* ≥ 5).

To further analyze the importance of APN dynamics, we engineered disulfide bonds to bridge domains II and IV and restrict ectodomain motion. We replaced the pAPN domain II Ser464 and domain IV Ser892 and/or Ser929 with cysteine to lock the ectodomain in the closed form with interdomain disulfide bridges. The Ser464 main chain Cα is ~5 and ~6 Å, respectively, from Ser892 and Ser929 in the closed form, but Ser464 moves ~12 Å away in the open form (Supplementary Fig. S4). Disulfide bond formation between pAPN Cys464 and Cys892 or Cys929 should thus prevent ectodomain motion. We expressed the pAPN-cysteine mutants (C2-C4) on the 293T cell surface and compared their catalytic and CoV binding activity with that of the wild type pAPN (Fig. 6).

The pAPN cysteine mutants showed reduced catalytic activity (Fig. 6a) and TGEV RBD binding (Fig. 6b) relative to the wild type protein in 293T transfectants that express similar protein amounts. The higher activity of the pAPN C2 than the C3 or C4 mutants suggested that the Cys464-Cys892 disulfide bond was more labile than the Cys464-Cys929 bond, probably because Cys892 is in a flexible loop (Supplementary Fig. S4). Treatment with a reducing agent restored catalysis and RBD binding in the cysteine mutants and did not affect wild type pAPN binding activity (Fig. 6). Reducing the disulfide bonds fully restored RBD binding, but catalysis was partially recovered in the pAPN C3 and C4 mutants. Substrate hydrolysis is proposed to close the ectodomain (see above), which would facilitate rebuilding the disulfides. Locking the closed form and the phenylalanine in the domain IV ARM repeat inside the active site probably impeded substrate processing (Fig. 2a). The pAPN cysteine mutants bound markedly less RBD than the wild type protein, which confirmed that CoV S protein binding to the closed pAPN was sterically hindered (Fig. 2b), and that CoV recognized the open form. Overall, these results validate the functional relevance of the APN ectodomain conformations and its motion.

## Discussion

Structural dynamics is an intrinsic property of aminopeptidases. The APN crystal structures reported here indicate the dynamic conformation of its ectodomain, and functional studies show its relevance in catalysis and virus infection. Distinct ectodomain regions mediate these functions, but agents that bind to one region prevent activities linked to the other. These allosteric effects with ligands are probably caused by restrictions in APN conformational dynamics, as confirmed with disulfide bond mutants. They demonstrated that preventing ectodomain motion and locking APN forms inhibits its functions.

APN ectodomain movement is less pronounced and differs from that reported for other M1 aminopeptidases. These differences could be due to the APN dimeric conformation and its linkage to the cell surface. Dimerization only engages the domain IV region, and we found that the dimer is conserved in all APN structures, closed, intermediate and open. APN domain IV thus does not move as described for ERAP-1 or F3^23^,^24^, proteins that do not form dimers. The fixed conformation of the APN dimer determines that the domain I to III module swings over domain IV (Supplementary Video S1), with the hinge at the domain IV N-terminal region. The length of this movement is less marked in APN (15°) than in ERAP-1 (22°), although the two proteins have very similar closed conformations. Displacement of the APN domains I, II and III must be limited by the length of the flexible polypeptide that links domain I to the transmembrane region, whose movement is restricted by membrane fluidity. The extent of APN movement nonetheless appears to be sufficient for release of the hydrolyzed peptide N-terminal residue, which is not plugged by domain IV in the open or in the intermediate APN conformations (Fig. 2a). It is not clear how each monomer in the dimer moves, whether their movement is random or synchronized in the same or inverse directions. Experiments with hAPN antibodies^25^ and those shown here with the TGEV RBD (Fig. 3) suggest that ~50% of the molecules adopt different forms; these data imply that each APN monomer maintains a distinct conformation (Supplementary Video S1).

The crystal structures reported here provide snapshots of APN dynamic conformation, and also guided experiments that demonstrate its role in virus entry into cells and catalysis. The switch between a proteolytic active (closed) and an inactive (open) conformation has been proposed for several M1 aminopeptidases^20^,^23^,^24^,^28^. This dynamics is thought to be important for peptide hydrolysis and release from the aminopeptidase active site. The region that joins α26 and α27 in the domain IV ARM repeat penetrates the active site groove in closed pig and human APN structures reported here and elsewhere (Fig. 2a)^19^, and a conserved phenylalanine in this region locks the substrate coordinated to the zinc ion, permitting hydrolysis. Further peptide processing likely requires removal of the phenylalanine lock by opening the APN ectodomain, which facilitates N-terminal residue release and peptide translocation, both sterically hindered in the closed conformation (Fig. 2a). The inherent flexibility in the domain IV ARM repeat that we demonstrate here is linked to interdomain arrangements might also enable substrate processing, and indicate how ectodomain movements participate in peptide hydrolysis. Local changes in a conserved tyrosine (Tyr472 in pAPN) at the active site of M1 aminopeptidases are also suggested to be important^20^,^24^,^28^. Among APN forms, the absence of conformational switches in active site residues at domain II (Supplementary Fig. S2) nonetheless indicates that tyrosine movement is not linked to interdomain motion.

Disulfide bonds that lock the APN closed conformation or drugs that prevent opening of the ectodomain inhibited CoV protein binding and cell infection, whereas porcine CoV S proteins probably hinder APN transition to the closed form and peptide hydrolysis. Our results verify the critical role of APN dynamics in CoV infection and catalysis, and demonstrate that the open APN structure is inactive in peptide hydrolysis. Anti-APN antibodies that inhibit APN activity and reduce tumor growth^25^,^33^ likely block ectodomain movements. The allosteric inhibition of APN functions shown here using viral proteins and drugs is likely to be due to suppression of APN transient conformational states, as shown for other enzymes^34^. Blocking APN movement prevents its functions, and suggests a new approach for the development of drugs that target this protein. Small molecules or conformation-specific antibody inhibitors of ectodomain motions can bind to the active site or interact with distant sites, as shown here with CoV spike fragments. High affinity drugs designed to inhibit catalysis and tumor growth prevent CoV infections, which indicates that targeting APN ectodomain dynamics can be a valuable approach to block APN functions related to cancer progression and virus infections.

## Methods

### Recombinant proteins

The engineered hAPN and pAPN ectodomains contain extracellular residues 36 to 967 and 36 to 963, respectively. They have a hemagglutinin (HA; YPYDVPDYA) peptide at the N terminus. Soluble ectodomains were produced in stably transfected CHO-Lec 3.2.8.1 (CHO-Lec) cells with selective culture media^35^. A pAPN protein with Met residues replaced by seleno-Met (Se-Met pAPN) was produced using methionine- and glutamine-free DMEM (Invitrogen) supplemented with 10% dialyzed fetal calf serum (FCS) and L-seleno-methionine (both from Sigma). APN proteins secreted to culture supernatants were purified by affinity chromatography with anti-HA 12AC5 mAb (Roche), followed by size exclusion chromatography in HEPES-saline buffer (20 mM HEPES, 150 mM NaCl) pH 7.5.

Preparation of most soluble CoV S proteins used here has been described^16^,^35^. S1H and S3H proteins were derived from the HOL87 porcine CoV S glycoprotein and bear the pAPN-binding domain. Soluble TGEV RBD was derived from the S glycoprotein of the TGEV SC11 strain (GenBank acc. n° AJ271965). It contains S residues 505 to 657, an N-terminal HA peptide, and a FLAG sequence (monovalent RBD variant) or human IgG1 Fc (bivalent RBD-Fc variant) at the C-terminal end. CoV S proteins were produced in CHO-Lec or 293 T cells and purified as described^35^.

### Analysis of APN catalysis

APN catalytic activity was determined using leucine p-nitroanilide (L-pNA) (Sigma) in a standard spectrophotometric assay in 96-well plates with soluble proteins or transfected cells.

To study CoV protein inhibition of APN catalysis, soluble APN ectodomains (5 μg/ml; ~40 nM) were added to duplicate wells, alone or with soluble CoV S protein variants, followed by the L-pNA substrate (1 mM) in 100 μl final volume (4 °C). Plates were incubated at room temperature and OD at 405 nm was measured at different times. Background OD of wells without APN was subtracted to determine specific catalytic activity. Similar procedure was used with 293T cells (3 × 10^4^) expressing pAPN 36 hr after transfection. OD of well with mock-transfected cells were taken as background. Cell samples expressing various amounts of pAPN at the membrane were used to normalize the activity of the pAPN cysteine mutants. Relative activity of the mutant to wild type was determined as the ratio of the pAPN mutant to the wild type OD from samples with the same protein expression, as monitored by flow cytometry (see below).

### CoV protein binding to APN

Stably transfected BHK21-pAPN, CHO-pAPN and CHO-pAPN mutant cells or transiently transfected 293T cells were used. The pAPN contained the HA peptide at the C terminus to monitor cell surface expression in CHO and 293T cells. In the pAPN-HH/AA mutant, the two active site histidines were replaced with alanines, whereas in the pAPN cysteine mutants, the domain II Ser464 and the domain IV Ser892 and/or Ser929 were substituted by cysteine. We analyzed the effect on RBD binding to pAPN of two natural inhibitors of APN enzyme activity, bestatin and actinonin (Sigma)^29^, as well as four synthetic ABS compounds^30^. Bestatin and actinonin were dissolved at 25 mM in PBS, whereas ABS compounds were used at 20 mM in DMSO.

In wild type or mutant pAPN-expressing cells, we used flow cytometry to monitor TGEV RBD binding to cell surface pAPN, essentially as reported^16^,^35^. Cells were washed three times with cold PBS and resuspended (10^6^ cells/ml) in PBS supplemented with 0.3% heat-inactivated FCS and 0.125% bovine serum albumin (BSA; binding buffer); 200 μl of cell suspension were added to 96-well plates (Nunc), cells were sedimented and resuspended in 20 μl of 1–2 μg/ml RBD-Fc solution alone or with inhibitors at indicated concentrations (30 min, 4 °C). An unrelated Fc fusion protein was used as control. Cells were washed and incubated with anti-human IgG fluorescein isothiocyanate (FITC)-labeled secondary antibody (30 min, 4 °C). The mean fluorescent intensity was determined in a Beckman Coulter EPICS XL1; background cell staining with the Fc protein was subtracted to determine the specific RBD-Fc binding to cell surface pAPN. In parallel, the amount of cell surface pAPN expression was determined by flow cytometry with the anti-HA 12AC5 mAb (Roche) and an anti-mouse FITC-labeled secondary antibody (Invitrogen). Analysis of the pAPN cysteine mutants binding activity was normalized by the cell surface protein amounts as explained above for the catalytic activity.

### Inhibition of CoV cell entry and infection

TGEV cell entry was quantified by determination of viral RNA by qRT-PCR (quantitative reverse transcription polymerase chain reaction). Stable transfected BHK21-pAPN or BHK21 cells (5 × 10^4^ cells/well) in DMEM (Dulbecco’s modified Eagle’s medium) with 10% FCS were plated in 24-well plates (18 h). Plates were transferred to 4 °C, medium was removed and 200 μl binding buffer alone or with APN-binding drugs or RDB protein were added to wells; after 10 min, the solution was replaced with 200 μl virus inoculum at a multiplicity of infection (m.o.i) of 1, alone or with inhibitors in binding buffer. After virus adsorption at 4 °C, cells were washed three times with binding buffer, and incubated in DMEM with 5% FCS (6 h, 37 °C, 5% CO_2_). Cells were detached and lysed with 100 μl Tri Reagent (Sigma) for RNA extraction, and cDNA was generated from 1 μg RNA using the High Capacity cDNA Reverse Transcription Kit (Applied Biosystems). Real-time PCR reactions (10 μl) were performed in triplicate using 5 μl cDNA sample, 2 μl of 5x HOT FIREPol EvaGreen qPCR Mix Plus (ROX) (Solis Biodyne) and 0.3 μl of specific primers for mouse β-actin or for the TGEV S gene, in a 7500 Real Time PCR system (Applied Biosystems) using a standard protocol. Data were analyzed with 7500 Software using the Comparative Ct Method (ΔΔCt). TGEV S expression relative to β-actin was determined and the ratio of values alone and with inhibitor used as relative cell entry.

Infection or cytopathic effect of TGEV was inhibited in porcine ST cells. One day after seeding (2.5 × 10^4^ cells/well) in 96-well plates, cells were transferred to 4 °C and pre-incubated with 50 μl binding buffer alone or with inhibitors, in duplicate. Solutions were replaced with 50 μl of serial 10-fold dilutions of virus inoculum with inhibitors or with DMSO (≤0.25%) as control. After incubation (1 h, 4 °C), cells were washed three times with DMEM with 5% FCS and incubated alone or with inhibitors for two days at 37 °C. To determine cell survival after infection, medium was removed, cells were formalin-fixed, stained with crystal violet and viability determined by optical density (OD) at 590 nm. Ratios from wells with and without virus were determined to calculate cell survival (see Supplementary Fig. S3b).

### Crystallization and diffraction data collection

The endoglycosidase H-treated (16 h, 30 °C) pAPN (pAPNeh) ectodomain was crystallized by the sitting drop technique with a crystallization solution of 5% polyethylene glycol (PEG)-1000 and 10% PEG-8000 (pH ~6) and a 15 mg/ml protein sample. Alternatively, native glycosylated pAPN ectodomain crystals were prepared with a crystallization solution of 20% PEG-3350 and 100 mM sodium acetate pH 5.6. The hAPN ectodomain (15 mg/ml) was crystallized with a solution of 20% PEG-6000, 50 mM imidazole-HCl pH 8.0. Crystals were frozen in crystallization solutions containing 20% ethylene glycol for diffraction data collection at the European Synchrotron Radiation Facility (ESRF; ID14 and ID23) and Swiss Light Source (SLS; PXII) beamlines. Diffraction data were processed with XDS^36^ and scaled with SCALA programs^37^. For statistical data, see Table 1.

### Structure determination

The structure of Se-Met pAPNeh protein was solved by a combination of molecular replacement (MR) and single-wavelength anomalous dispersion (SAD) methods. The crystals contained two molecules in the asymmetric unit (Table 1). A partial structure was obtained by MR using the PHASER program^38^ and domains I to III of the tricorn-interacting factor F3 (PDB code 1Z1W), which share ~30% residue identity with pAPN. The PHASER LLG value for the best MR solution was 161, whereas RFZ values were 6.5 and 5.0, and TFZ values of 3.0 and 9.9. We then used the MRSAD protocol in the Auto-Rickshaw server^39^ to determine the complete pAPNeh structure, starting from the partial MR structure and using Se-Met pAPNeh crystal diffraction data collected at the selenium peak wavelength. The final structure included the two pAPN molecules of the asymmetric unit, which were adjusted manually and refined with phenix.refine^40^ using data extending to 2.5 Å resolution (for statistics, see Table 1). The pAPNeh structure comprises residues 60 to 963 and the zinc atoms coordinated in the enzyme active site.

The other APN ectodomain structures (Table 1) were determined by the MR method using the pAPNeh structure as search model. Two ensembles including domain I, II and III or isolated domain IV were used for MR structure determination with PHASER. Structures were refined with phenix.refine (statistics in Table 1). In all structures, the engineered tags and 25–30 residues of the N-terminal ectodomains were very disordered and are not included in the final models. Electron density maps of active site residues and of N-linked glycans are shown in Supplementary Figs S2 and S5, respectively. Structure representations prepared with PyMOL (pymol.org).

## Additional Information

**How to cite this article:** Santiago, C. *et al*. Allosteric inhibition of aminopeptidase N functions related to tumor growth and virus infection. *Sci. Rep.* **7**, 46045; doi: 10.1038/srep46045 (2017).

**Publisher's note:** Springer Nature remains neutral with regard to jurisdictional claims in published maps and institutional affiliations.

## Supplementary Material

Supplementary Information

Supplementary Video S1

## Figures and Tables

**Table 1:** Data Collection and Refinement Statistics.

	Se-Met pAPNeh	pAPN	hAPN
Data Collection			
Space group	P2_1_	P1	P2_1_2_1_2_1_
Cell dimensions			
a, b, c (Å)	66.5, 215.7, 78.6	78.7, 78.8, 223.9	127.1, 168.9, 244.2
α, β, γ (°)	90.0, 91.9, 90.0	99.7, 92.6, 111.3	90.0, 90.0, 90.0
Resolution (Å)	25–2.5 (2.64)	25–2.0 (2.11)	20–2.6 (2.74)
Unique reflections	73986	322701	160355
Redundancy	4.1 (3.9)	2 (2.0)	4.2 (4.1)
Completeness (%)	97 (95.5)	97.5 (96.6)	99.4 (99.8)
R_sym_ or R_merge_	10.5 (35.6)	7.5 (20.7)	5 (35.3)
I/s(I)	6 (1.8)	8.5 (3.6)	12.3 (2.1)
CC(1/2)	0.986 (0.854)	0.981 (0.927)	0.999 (0.934)
Refinement			
Resolution (Å)	25.0–2.5 (2.56)	25.0–2.0 (2.02)	20–2.6 (2.63)
R_work_	19.0 (23.6)	17.1 (19.8)	18.1 (27.6)
R_free_	22.9 (28.8)	20.6 (25.0)	21.4 (32.3)
No. of atoms			
Protein	14292	28817	29182
Carbohydrates	252	726	1417
Ligands	—	16	—
Ion (Zn)	2	4	4
Water	381	4899	707
Average B-factors			
Protein	20	13	55
Carbohydrates	37	32	101
Ligands	—	11	—
Ions (Zn)	17	6	43
Water	20	26	47
R.m.s deviations			
Bond lengths (Å)	0.009	0.009	0.004
Bond angles (°)	1.28	0.977	0.77
Ramachandran plot	96.0/3.5/0	96.6/3.4/0	96.6/3.4/0
Molecules asu	2	4	4
PDB code	5LG6	5LDS	5LHD

Statistics for the structures of the native and endoglycosidase H-treated porcine APN (pAPN and pAPNeh) and of the human APN (hAPN) proteins. The Met residues of the pAPNeh were replaced by seleno-Met (see Methods). Highest-resolution shell is in parentheses. Favored, allowed and outlier residues (%) in the Ramachandran plot, as well as number of ectodomains in the asymmetric unit (asu) are shown. Statistics for the pAPN-RBD crystal structure discussed here have been reported earlier (PDB code 4F5C)^16^. Structure representations in Supplementary Fig. 1b.

**Table 2:** Characteristics of the APN structures.

Structure	pAPN	hAPN	pAPN-RBD
Conformation	closed	intermediate	open
Intermonomer distance	95 Å	116 Å	131 Å
Interdomain surfaces (Å^2^)			
DI-DII	1732	1729	1686
DII-DIII	1341	1329	1243
DIII-DIV	1161	1121	1016
DIV-DI	163	8	—
DIV-DII	1754	1126	754
DIV-DIV	939	928	981
Interdomain angles	0	6°	15°

Intermonomer distances in the APN dimers were determined between the N-terminal residues of first β-strands. Interdomain buried surfaces in the crystal structures were computed with the PISA server (http://www.ebi.ac.uk/msd-srv/prot_int/cgi-bin/piserver). The domain IV buried surface in each monomer at the dimer interface is shown as DIV-DIV. Interdomain angles of the open and intermediate structures were determined relative to the closed pAPN after structure superposition based on domain IV. Angles were computed based on the zinc ions at domain II and the hinge residue at the domain IV N terminus with the COOT program^41^. The pAPNeh structure has very similar conformation to the native pAPN.

## References

[b1] Taylor A. Aminopeptidases: structure and function. FASEB J. 7, 290–298 (1993). 8440407 10.1096/fasebj.7.2.8440407

[b2] Hooper N. M. Families of zinc metalloproteases. FEBS Lett. 354, 1–6 (1994). 7957888 10.1016/0014-5793(94)01079-x

[b3] Mina-Osorio P. The moonlighting enzyme CD13: old and new functions to target. Trends Mol. Med. 14, 361–371 (2008). 18603472 10.1016/j.molmed.2008.06.003 PMC7106361

[b4] Delmas B. . Aminopeptidase N is a major receptor for the entero-pathogenic coronavirus TGEV. Nature 357, 417–420 (1992). 1350661 10.1038/357417a0 PMC7095137

[b5] Yeager C. L. . Human aminopeptidase N is a receptor for human coronavirus 229E. Nature 357, 420–422 (1992). 1350662 10.1038/357420a0 PMC7095410

[b6] Fujii H. . Human melanoma invasion and metastasis enhancement by high expression of aminopeptidase N/CD13. Clin. Exp. Metastasis 13, 337–344 (1995). 7641419 10.1007/BF00121910 PMC7088232

[b7] Pasqualini R. . Aminopeptidase N is a receptor for tumor-homing peptides and a target for inhibiting angiogenesis. Cancer Res. 60, 722–727 (2000). 10676659 PMC4469333

[b8] Sato Y. Role of aminopeptidase in angiogenesis. Biol Pharm Bull 27, 772–776 (2004). 15187415 10.1248/bpb.27.772

[b9] Danziger R. S. Aminopeptidase N in arterial hypertension. Heart Fail. Rev. 13, 293–298 (2008). 18008160 10.1007/s10741-007-9061-y PMC7088157

[b10] Wickström M., Larsson R., Nygren P. & Gullbo J. Aminopeptidase N (CD13) as a target for cancer chemotherapy. Cancer Science 102, 501–508 (2011). 21205077 10.1111/j.1349-7006.2010.01826.x PMC7188354

[b11] Rangel R. . Impaired angiogenesis in aminopeptidase N-null mice. Proc. Natl. Acad. Sci. USA 104, 4588–4593 (2007). 17360568 10.1073/pnas.0611653104 PMC1815469

[b12] Corti A., Curnis F., Arap W. & Pasqualini R. The neovasculature homing motif NGR: more than meets the eye. Blood 112, 2628–2635 (2008). 18574027 10.1182/blood-2008-04-150862 PMC2556602

[b13] Inagaki Y. . Novel aminopeptidase N (APN/CD13) inhibitor 24F can suppress invasion of hepatocellular carcinoma cells as well as angiogenesis. Biosci. Trends 4, 56–60 (2010). 20448342

[b14] Masters P. S. The molecular biology of coronaviruses. Adv. Virus Res. 66, 193–292 (2006). 16877062 10.1016/S0065-3527(06)66005-3 PMC7112330

[b15] Tusell S. M., Schittone S. A. & Holmes K. V. Mutational analysis of aminopeptidase N, a receptor for several group 1 coronaviruses, identifies key determinants of viral host range. J. Virol. 81, 1261–1273 (2007). 17093189 10.1128/JVI.01510-06 PMC1797531

[b16] Reguera J. . Structural Bases of Coronavirus Attachment to Host Aminopeptidase N and Its Inhibition by Neutralizing Antibodies. PLoS Pathog. 8, e1002859, 10.1371/journal.ppat.1002859 (2012). 22876187 PMC3410853

[b17] Danielsen E. M. Biosynthesis of intestinal microvillar proteins. Dimerization of aminopeptidase N and lactase-phlorizin hydrolase. Biochemistry 29, 305–308 (1990). 1969748 10.1021/bi00453a042

[b18] Sjostrom H., Noren O. & Olsen J. Structure and function of aminopeptidase N. Adv Exp. Med. Biol. 477, 25–34 (2000). 10849727 10.1007/0-306-46826-3_2

[b19] Wong A. H., Zhou D. & Rini J. M. The X-ray crystal structure of human aminopeptidase N reveals a novel dimer and the basis for peptide processing. J. Biol. Chem. 287, 36804–36813 (2012). 22932899 10.1074/jbc.M112.398842 PMC3481283

[b20] Addlagatta A., Gay L. & Matthews B. W. Structure of aminopeptidase N from *Escherichia coli* suggests a compartmentalized, gated active site. Proc. Natl. Acad. Sci. USA 103, 13339–13344 (2006). 16938892 10.1073/pnas.0606167103 PMC1569165

[b21] Chen L., Lin Y.-L., Peng G. & Li F. Structural basis for multifunctional roles of mammalian aminopeptidase N. Proc. Natl. Acad. Sci. USA 109, 17966–17971 (2012). 23071329 10.1073/pnas.1210123109 PMC3497818

[b22] Reguera J., Mudgal G., Santiago C. & Casasnovas J. M. A structural view of coronavirus-receptor interactions. Virus Res. 194, 3–15 (2014). 25451063 10.1016/j.virusres.2014.10.005 PMC7114462

[b23] Kyrieleis O. J. P., Goettig P., Kiefersauer R., Huber R. & Brandstetter H. Crystal Structures of the Tricorn Interacting Factor F3 from Thermoplasma acidophilum, a Zinc Aminopeptidase in Three Different Conformations. J. Mol. Biol. 349, 787–800 (2005). 15893768 10.1016/j.jmb.2005.03.070

[b24] Kochan G. . Crystal structures of the endoplasmic reticulum aminopeptidase-1 (ERAP1) reveal the molecular basis for N-terminal peptide trimming. Proc. Natl. Acad. Sci. USA 108, 7745–7750 (2011). 21508329 10.1073/pnas.1101262108 PMC3093473

[b25] Xu Y., Wellner D. & Scheinberg D. A. Cryptic and regulatory epitopes in CD13/aminopeptidase N. Exp. Hematol. 25, 521–529 (1997). 9197331

[b26] Su L. . Development of Synthetic Aminopeptidase N/CD13 Inhibitors to Overcome Cancer Metastasis and Angiogenesis. ACS. Med. Chem. Lett. 3, 959–964 (2012). 24900417 10.1021/ml3000758 PMC4025871

[b27] Schmitt C. . Selective aminopeptidase-N (CD13) inhibitors with relevance to cancer chemotherapy. Bioorg. Med. Chem. 21, 2135–2144 (2013). 23428964 10.1016/j.bmc.2012.12.038

[b28] Nguyen T. T. . Structural basis for antigenic peptide precursor processing by the endoplasmic reticulum aminopeptidase ERAP1. Nat. Struct. Mol. Biol. 18, 604–613 (2011). 21478864 10.1038/nsmb.2021 PMC3087843

[b29] Bauvois B. & Dauzonne D. Aminopeptidase-N/CD13 (EC 3.4.11.2) inhibitors: chemistry, biological evaluations, and therapeutic prospects. Med. Res. Rev. 26, 88–130 (2006). 16216010 10.1002/med.20044 PMC7168514

[b30] Maiereanu C. . A novel amino-benzosuberone derivative is a picomolar inhibitor of mammalian aminopeptidase N/CD13. Bioorg. Med. Chem. 19, 5716–5733 (2011). 21843945 10.1016/j.bmc.2011.06.089

[b31] Delmas B. . Determinants essential for the transmissible gastroenteritis virus-receptor interaction reside within a domain of aminopeptidase-N that is distinct from the enzymatic site. J. Virol. 68, 5216–5224 (1994). 7913510 10.1128/jvi.68.8.5216-5224.1994 PMC236465

[b32] Revelant G. . Exploring S1 plasticity and probing S1′ subsite of mammalian aminopeptidase N/CD13 with highly potent and selective aminobenzosuberone inhibitors. Bioorg. Med. Chem. 23, 3192–3207 (2015). 25982416 10.1016/j.bmc.2015.04.066

[b33] Akita S. . MT95-4, a fully humanized antibody raised against aminopeptidase N, reduces tumor progression in a mouse model. Cancer Sci. 106, 921–928 (2015). 25950387 10.1111/cas.12692 PMC4520645

[b34] Tzeng S.-R. & Kalodimos C. G. Allosteric inhibition through suppression of transient conformational states. Nat. Chem. Biol. 9, 462–465 (2013). 23644478 10.1038/nchembio.1250

[b35] Reguera J., Ordoño D., Santiago C., Enjuanes L. & Casasnovas J. M. Antigenic modules in the N-terminal S1 region of the Transmissible Gastroenteritis Virus spike protein. J. Gen. Virol. 92, 1117–1126 (2011). 21228126 10.1099/vir.0.027607-0 PMC3139418

[b36] Kabsch W. X. D. S. Acta Cryst. D66, 125–132 (2010). 10.1107/S0907444909047337 PMC2815665 20124692

[b37] Collaborative Computational Project, N. The CCP4 Suite: Programs for Protein Crystallography. *Acta Cryst.* D50, 760-763 (1994). 10.1107/S0907444994003112 15299374

[b38] Read R. J. Pushing the boundaries of molecular replacement with maximum likelihood. Acta Cryst. D57, 1373–1382 (2001). 10.1107/s0907444901012471 11567148

[b39] Panjikar S., Parthasarathy V., Lamzin V. S., Weiss M. S. & Tucker P. A. On the combination of molecular replacement and single-wavelength anomalous diffraction phasing for automated structure determination. Acta Cryst. D65, 1089–1097 (2009). 10.1107/S0907444909029643 PMC2756167 19770506

[b40] Adams P. D. . PHENIX: a comprehensive Python-based system for macromolecular structure solution. Acta Cryst. D66, 213–221 (2010). 10.1107/S0907444909052925 PMC2815670 20124702

[b41] Emsley P. & Cowtan K. Coot: model-building tools for molecular graphics. Acta Cryst. D60, 2126–2132 (2004). 10.1107/S0907444904019158 15572765

