# Supplementary material for: Allosteric inhibition of aminopeptidase N functions related to tumor growth and virus infection
Source: Sci Rep. 2017 Apr 10;7:46045. doi: 10.1038/srep46045 (PMC5385526; doi:10.1038/srep46045)
Supplement: Supplementary Information [file srep46045-s1.pdf]

Supplementary Materials

Allosteric inhibition of aminopeptidase N functions related to tumor growth and virus infection

César Santiago[1], Gaurav Mudgal[1,3], Juan Reguera[1,4], Rosario Recacha[1,5], Sébastien Albrecht[2], Luis Enjuanes[1] and José M Casasnovas[1,*]

[1]Centro Nacional de Biotecnología (CNB-CSIC), Darwin 3, Campus Universidad Autónoma de Madrid, 28049 Madrid, Spain

[2]Laboratoire de Chimie Organique et Bioorganique, Ecole Nationale Supérieure de Chimie Mulhouse, Université Haute-Alsace, 68093 Mulhouse, France

Present addresses:

[3]Department of Biotechnology, Institute of Engineering and Technology, Mangalayatan University, 33rd Milestone, Beswan, Aligarh, UP, India-202145

[4]AFMB Architecture et Fonction des Macromolécules Biologiques, CNRS Aix-Marseille University, 163 avenue de Luminy, 13288 Marseille Cedex 9, France

[5]Latvian Institute of Organic Synthesis, Aizkraukles 21, Riga LV-1006, Latvia

[*] jcasasnovas@cnb.csic.es

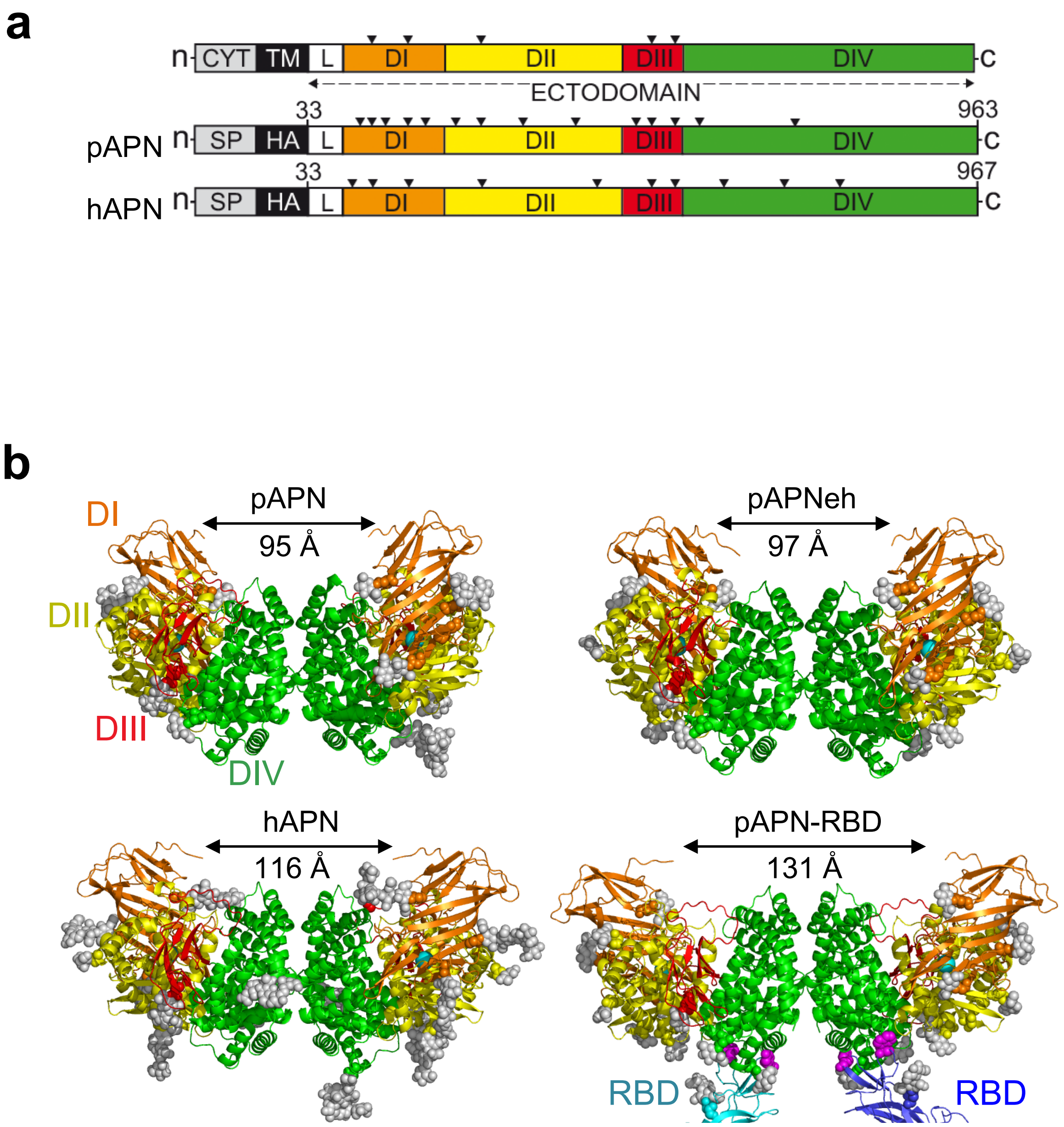

Supplementary Figure S1. Crystal structures of mammalian APN ectodomains. (*a*) Diagram of the APN modular structure (top) and the crystallized soluble ectodomains of the porcine (pAPN) and human (hAPN) proteins. The most N-terminal cytoplasmic (CYT) and transmembrane (TM) domains of the APN were replaced with a signal peptide (SP) and an HA epitope for expression of soluble ectodomains (see Methods). Colored boxes indicate domains I to IV. Flexible polypeptide (L) links domain I to the TM domain (white). N-glycosylation sites (black triangles). (*b*) Ribbon

diagrams of the dimeric APN ectodomain structures reported here and that of the pAPN in complex with the RBD of a porcine CoV (PDB code 4F5C) [16]. Structures of the native pAPN and hAPN ectodomains as well as the endoglycosidase H-treated pAPN (pAPNeh) are shown. APN domains (DI-DIV) colored as in a. Asn residues in N-linked glycosylation sites and modeled glycans (grey) are shown as spheres. Zinc metal ions at domain II active site shown as cyan spheres. The distance between the N-terminal residues of the first β-strand at domain I are indicated above each structure. The tips of the APN-bound RBD are shown in cyan and blue, with two key virus residues (Trp and Tyr) as magenta spheres.

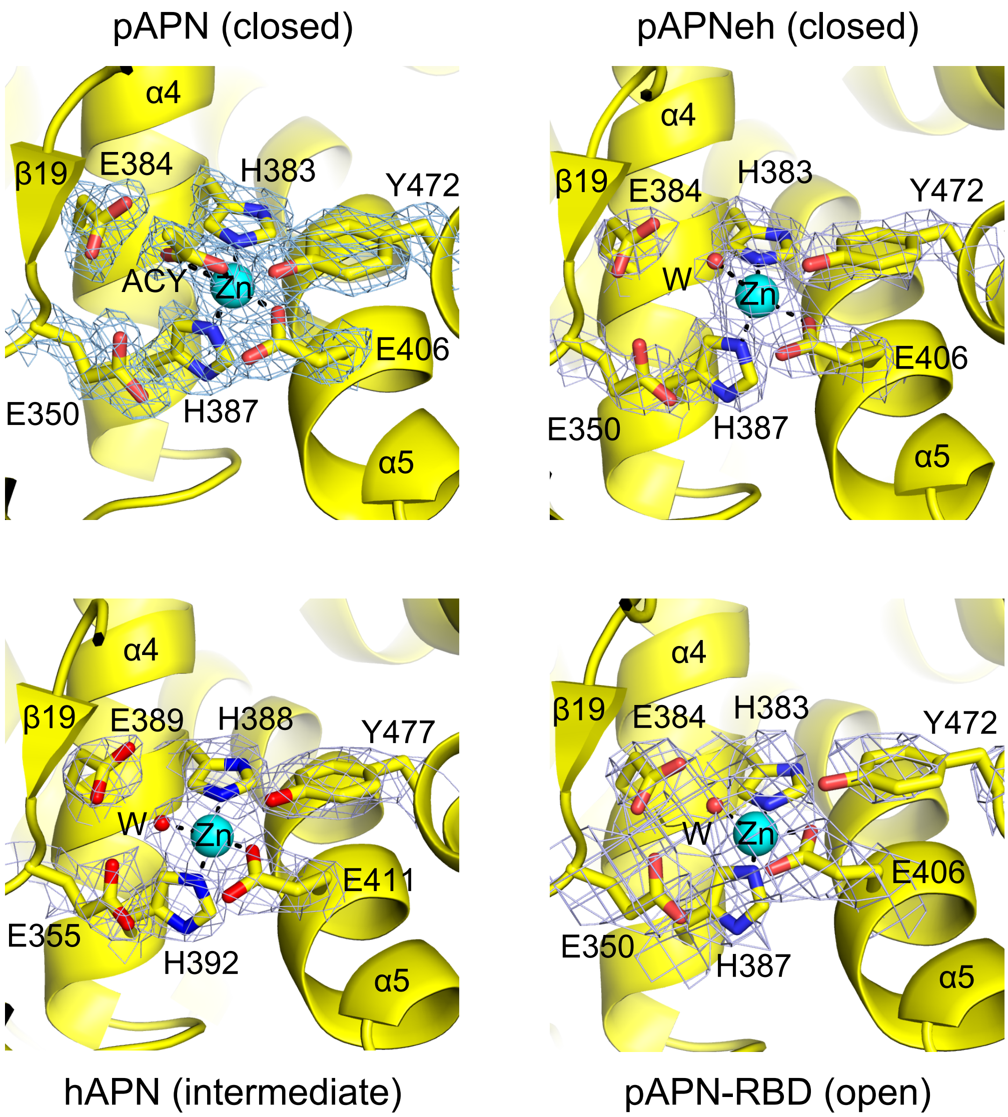

Supplementary Figure S2. The active site in the APN ectodomains. Ribbon diagram of the catalytic site of the four APN structures shown in Supplementary Fig. S1b. Domain II residue side chains involved in catalysis are shown as sticks and the coordinated zinc ion as a cyan sphere. An acetate (ACY) was coordinated to the zinc in the pAPN structure, whereas the other structures had a water molecule (W, red sphere) that is involved in hydrolysis of the N-terminal residue of peptide substrates [21]. Electron density (2Fo-Fc) maps are shown as a light blue mesh contoured at 1.5 sigma.

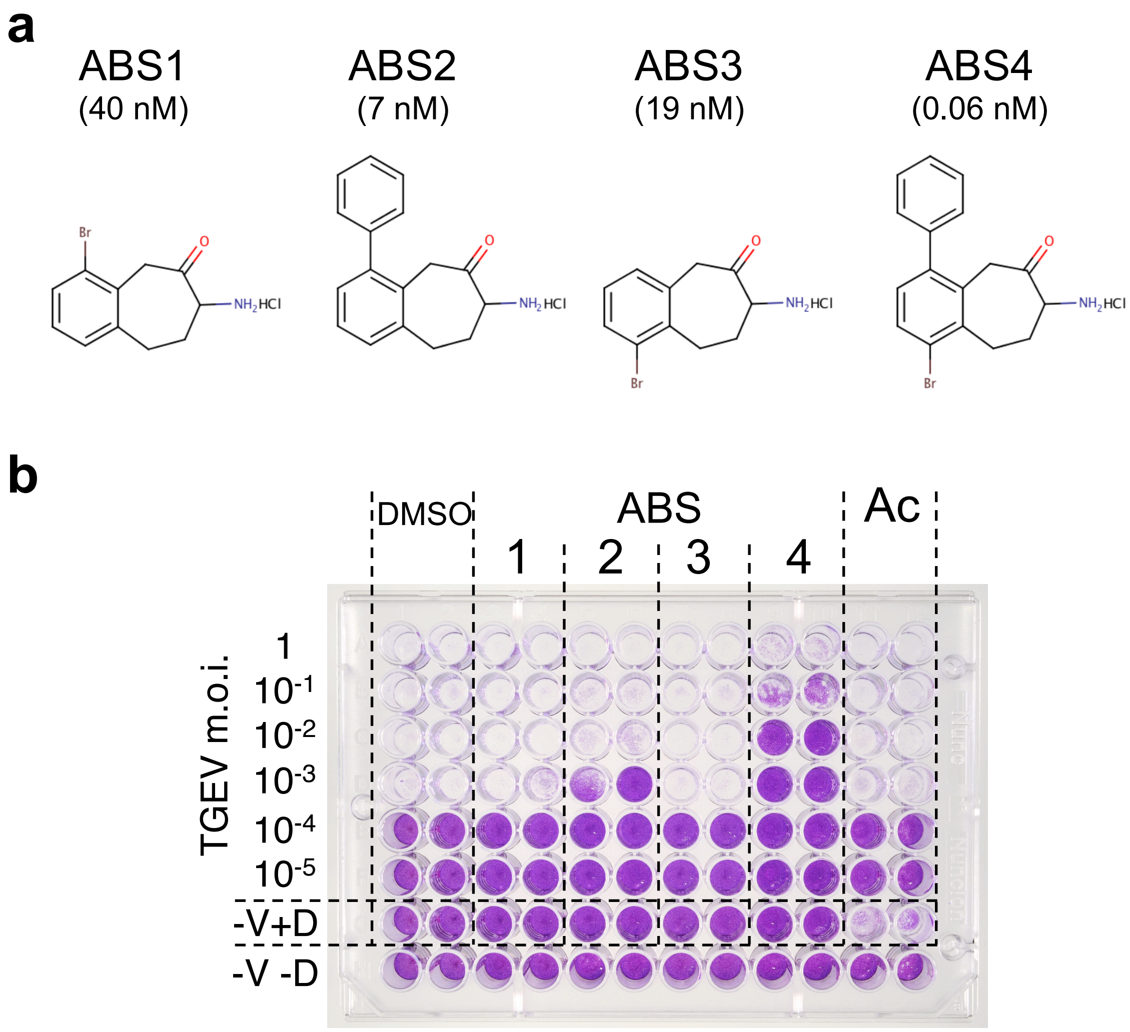

Supplementary Figure S3. Amino-benzosuberone (ABS) molecules and inhibition of CoV infectivity. (*a*) Diagram of the active site-binding ABS molecules, with the APN-binding affinity in parentheses [30]. (*b*) ABS compound inhibition of TGEV-mediated cytopathic effect in permissive ST cells. Stained cell monolayers in 96-well plates 48 h post-infection, with the indicated TGEV multiplicity of infection (m.o.i.), or uninfected cells with (-V+D) or without (-V-D) drug. Infection was carried out alone (-) or with ABS1-4 or actinonin (Ac) at 50 μM. Representative experiment; quantification in Fig. 5.

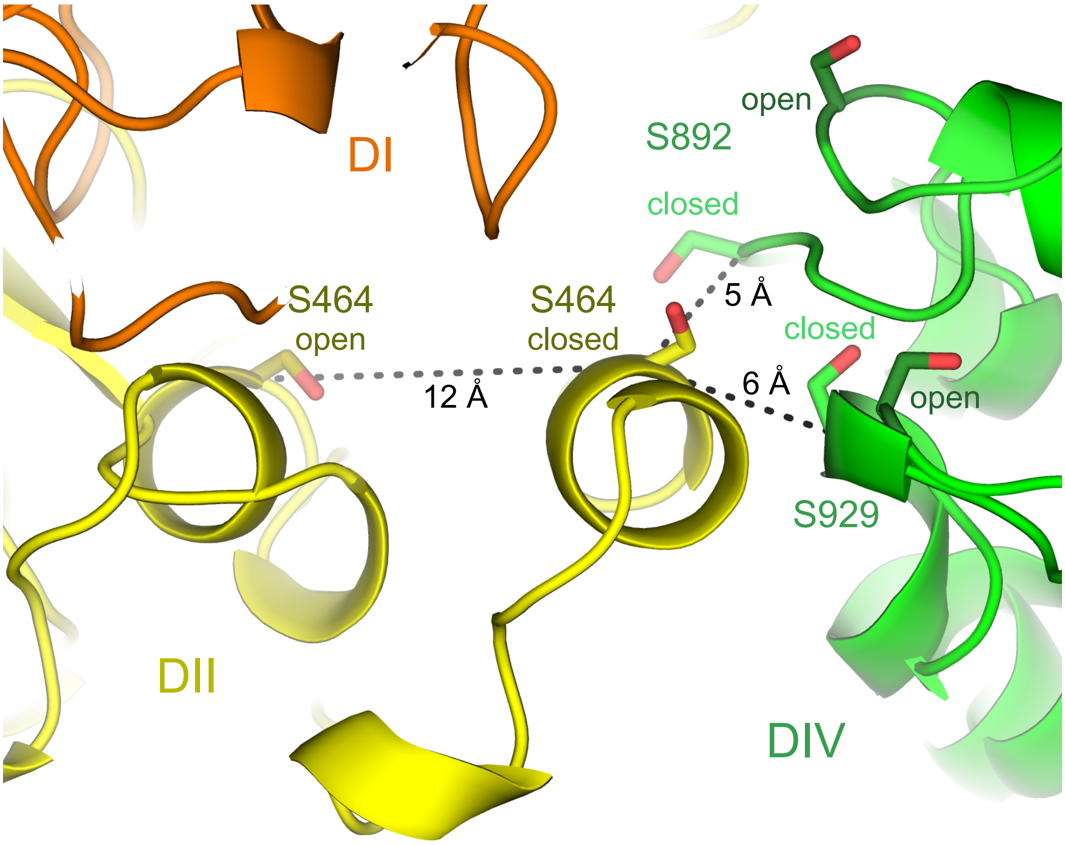

Supplementary Figure S4. Engineering disulfide bonds to bridge domain II and IV and restrict ectodomain motions. Ribbon representation of the pAPN open and closed structures superposed based on domain IV. Displacement of the pAPN domain II Ser464 (yellow) with respect to domain IV (green) between the closed and open forms. Ser464, and domain IV Ser892 and Ser929 were substituted by cysteine to lock the ectodomain in the closed form with interdomain disulfide bonds. Residue side chains and distances between their main chains Cα are shown. Residues in the open form are darker. Oxygens, red.

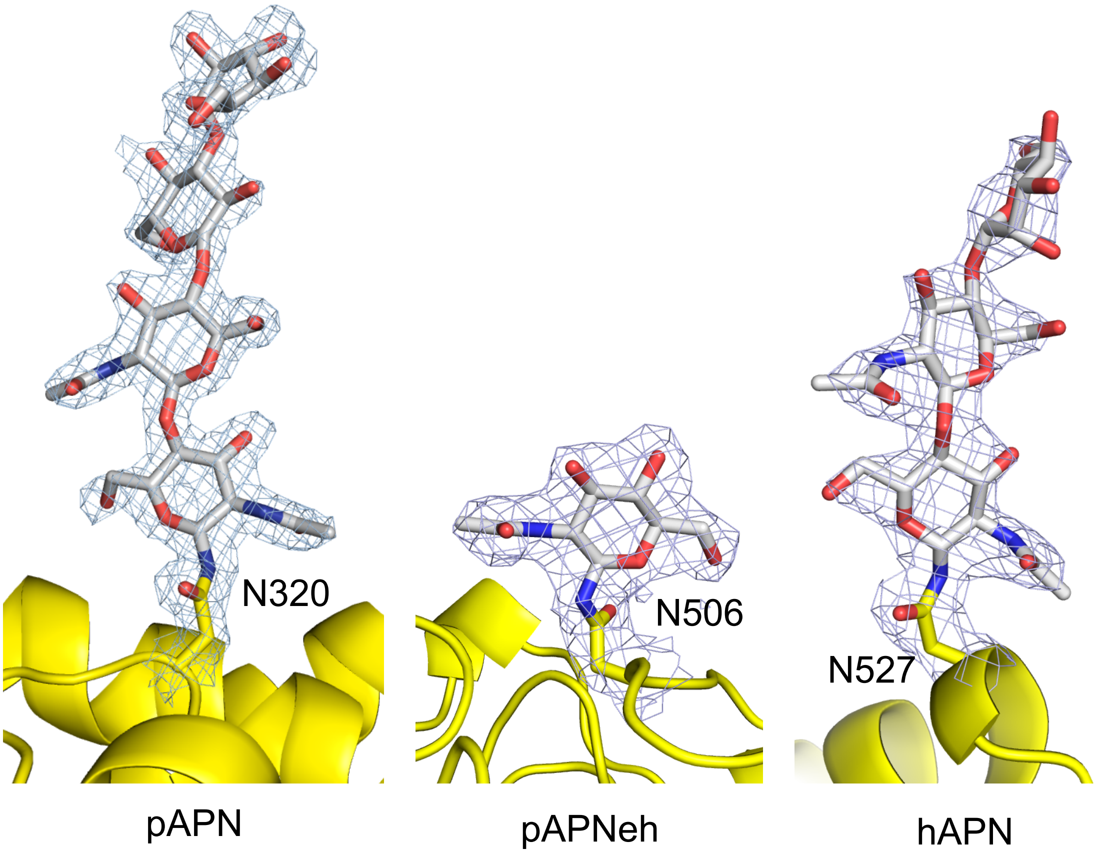

Supplementary Figure S5. N-linked glycosylations in the APN crystal structures. Ribbon diagrams of the APN domain II (yellow) with a well-defined glycan N-linked to the indicated asparagine in the three determined crystal structures (Table 1). The side chains of the asparagines (carbons in yellow) and the linked carbohydrate residues (carbons in grey) are shown with sticks and with the electron density (2Fo-Fc) maps, shown as a blue mesh contoured at 1 sigma. Nitrogens, blue; oxygens, red. The endoglycosidase H-treated pAPNeh protein only contained a N-acetylglucosamine.

Supplementary Video S1. Ectodomain dynamics in the dimeric APN. Movements computed throughout the transition between the open and closed APN states defined by the crystal structures. The dimer is preserved during movement of the ectodomain monomers. Ribbon representation with domains colored as in Fig. 1 and the zinc metal ions at domain II active site shown as cyan spheres; the side chain of the phenylalanine that penetrates into the active site in the closed conformation as dark-green sticks (Fig. 2a). Prepared with CHIMERA (http://www.cgl.ucsf.edu/chimera/).
